# Systematic study of resonant transmission effects in visible band using variable depth gratings

**DOI:** 10.1038/s41598-019-51414-3

**Published:** 2019-10-17

**Authors:** Andrei A. Ushkov, Alexey A. Shcherbakov, Isabelle Verrier, Thomas Kampfe, Yves Jourlin

**Affiliations:** 1 Univ Lyon, UJM-Saint-Etienne, CNRS, Institut d’Optique Graduate School, Laboratoire Hubert Curien UMR 5516, F-42023 Saint-Etienne, France; 2 ITMO University, Saint-Petersburg, 190000 Russia

**Keywords:** Optical materials and structures, Nanophotonics and plasmonics

## Abstract

The article focuses on depth-dependent visible band transmission effects in a symmetrical “insulator-metal-insulator” diffraction system based on a variable depth grating. These effects were studied both experimentally and theoretically in TM and TE polarizations. In particular, the existence of an optimized grating depth for plasmon-mediated resonant transmission was confirmed experimentally, and differences in TE and TM transmission behavior are discussed. We utilize a simple and flexible fabrication approach for rapid synthesis of apodized structures with adiabatically varying depth based on a beat pattern of two interferential lithography exposures. The present study can be useful in the fields of transmission-based optical security elements and biosensors.

## Introduction

Metal-dielectric diffraction gratings and metasurfaces supporting surface plasmon resonance (SPP) modes possess numerous attractive features for various optical applications including sensorics^1,2^, photovoltaics^3,4^, and security^5–7^. The functionality of these elements is commonly based on a strong coupling between propagating, waveguide, localized and surface modes^8–10^. The enhanced transmission effect^11–13^ or excitation of ultra-narrow resonances due to the bound states in the continuum^14^ are remarkable examples of resonant mode interactions which were profoundly studied for various types of periodic and non-periodic structures. Due to their resonant nature, the mentioned effects are strictly bound to some particular structure geometries and material parameters. An extension of the functionality of resonant gratings and metasurfaces often requires the development of multi-wavelength devices. A need in coupling the resonant behavior with broadband features leads to multidimensional structures like absorbers of anisotropic metamaterials with wavelength-scale structuring^15^ or wide band radiation trapping by graded depth lamellar gratings^16,17^. Due to multidimensionality the fabrication process of this kind of structures is inevitably split into several steps and may incorporate different technological processes, which makes the sample preparation quite a resource and time-consuming procedure.

Concerning the modulation of periodic structures which are adiabatic relative to the wavelength and periods, different approaches were proposed to achieve various functionalities. The first class of adiabatic modulations include the variation of shape and duty factor, which were adapted, for example, to develop high-efficient planar waveguide couplers^18–20^, to attain the “rainbow” trapping effect^16,17^, or to create effectively graded index planar functional optical layers^21^. The demonstrated structures were fabricated using ion and e-beam lithography in conjunction with mask printing. Variable dose e-beam writing is a technology that allows creating highly resolved microstructures with variable height at the cost of beeing very expensive and needing very long writing times^22^. There are alternative methods based on interference lithography, which utilize the superposition principle to yield optically variable gratings for security applications^23–26^. The technology is cheap and allows fabricating complex grating shapes on the basis of different interference patterns providing an additional phase-locking is applied^27^.

In this work we focus on the study of multidimensional metallic gratings which support the resonant transmission effect. Resonances in grating-based optical elements substantially depend on the depth^28^, and in this work we have developed a simple and flexible fabrication technology which allows one to rapidly create apodized structures with adiabatically varying depth. The proposed approach is based on the beats effect and extends the applicability of the known interference approaches^24,25^. The spatial period of the depth variations along the structure can be easily tuned, reaching macroscopic dimensions (several cm) which allows to use unfocused light beams for structure characterization. Our method relaxes requirements imposed on laser interference lithography (LIL) setups for fabrication of apodized gratings^24^, and provides much greater fabrication flexibility in comparison with small angle prism-based approaches^29^.

The paper is organized as follows. Section 2 describes the fabrication of varying-depth gratings including a detailed explanation of the proposed technique and the fabrication steps. Section 3 demonstrates results of experimental transmission measurements which are explained by quantitative rationalizations and rigorous numerical simulations. Finally, Section 4 concludes the text.

## Fabrication of Variable Depth Gratings

### Variable depth gratings

The basic idea underlying the fabrication of variable depth grating is to employ the effect of beats in the resist layer in a two step exposure LIL process. Generally, the two-beam LIL is an established method well-adopted for synthesis of 1D and 2D perfectly periodic structures of constant depth. Its workflow offers a relatively cheap and fast method of nanostructuring^30^. Here we propose an improvement to the method allowing one to fabricate periodic structures of variable depth with minor changes in the standard lithography scheme.

**Figure 1:**
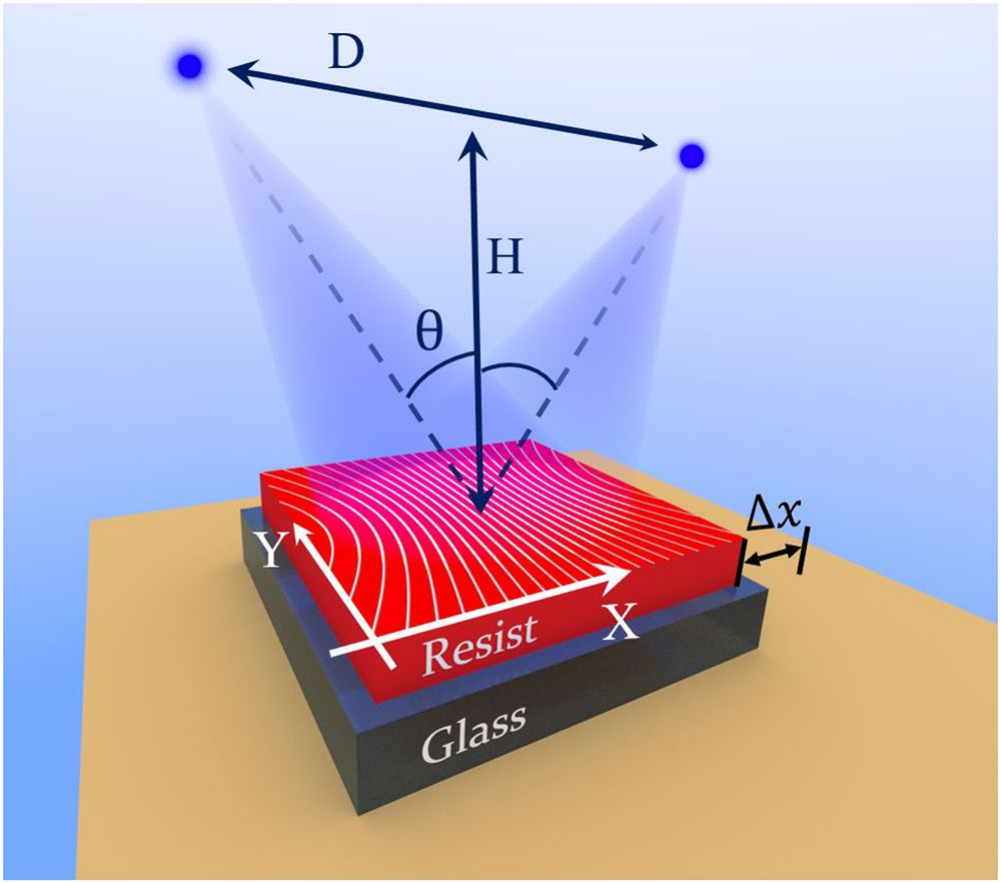
Scheme of LIL process with two coherent point light sources; *λ* = 442 nm (He-Cd laser), H = 32.3 cm, D = 69.3 cm, Δ*x* ~ 100 micron is controlled by linear translation stage. White lines on the resist surface represent the curved interference fringes (family of hyperbolae) in the X-Y plane.

In the conventional LIL technique a thin photoresist layer deposited on a substrate is being exposed by two coherent plane waves of wavelength *λ*, creating an interference pattern with a periodic variation of the light intensity inside the photoresist layer: *I*(*x*) = *I*_0_sin(2*πx*/Λ) + *I*_0_, with Λ = *λ*/2sin*θ* being the grating period that depends on the incidence angle *θ* of each beam. The next step in the LIL process consists in dissolving the resist in a developer. As the resist development rate depends monotonically on the absorbed energy dose^31^, the topology of the developed surface corresponds to the total intensity distribution of the previous exposition step. Figure 1 demonstrates a usual setup with two point sources, where the plane wave approximation is applicable in a relatively small zone far from the sources. One single exposure in LIL results in a 1D periodic surface undulation. In order to get more complex geometries several exposures can be applied. In case of two sequential LIL exposures of equal intensities *I*_0_ with periods Λ_1_ and Λ_2_, proposed e.g. in^24^, the total interference pattern is defined by the sum:

1 $$\begin{array}{rcl}I(x) & = & {I}_{0}\,\sin (\frac{2\pi }{{\Lambda }_{1}}x)+{I}_{0}\,\sin (\frac{2\pi }{{\Lambda }_{2}}x+\delta \phi )+2{I}_{0}\\  & = & 2{I}_{0}\,\sin (\frac{2\pi }{{\Lambda }_{1}{\Lambda }_{2}}\frac{{\Lambda }_{1}+{\Lambda }_{2}}{2}x+\frac{\delta \phi }{2})\cos (\frac{2\pi }{{\Lambda }_{1}{\Lambda }_{2}}\frac{{\Lambda }_{1}-{\Lambda }_{2}}{2}x+\frac{\delta \phi }{2})+2{I}_{0}\end{array}$$

*δφ* is a phase mismatch, which can be controlled only with a phase-locking scheme^27^, though its value is unimportant for our purpose as will be clarified later. If the periods are close to each other, Λ_1_ ≈ Λ_2_, and ΔΛ = |Λ_1_ − Λ_2_| ≪ Λ_1,2_, Eq. (1) can be simplified to:

2 $$I(x)\approx 2{I}_{0}\,\sin (\frac{2\pi }{{\Lambda }_{1}}x+\frac{\delta \phi }{2})\cos (\frac{2\pi }{2{\Lambda }_{1}^{2}/\Delta \Lambda }+\frac{\delta \phi }{2})+2{I}_{0}$$

According to Eq. (2) the total power distribution is modulated by the large scale period envelope Λ_*env*_ ≈ 2Λ_1_^2^/ΔΛ, while the small scale period is the same along the pattern and approximately equal to Λ_1_. Aiming at studying resonant effects in the visible band by depth resolved measurements on apodized structures, one should impose restrictions on both Λ_1_ and the degree of period proximity ΔΛ/Λ_1_. Estimating Λ ≡ Λ_1_ ≈ 300 nm and Λ_*env*_ ~ 1 cm gives ΔΛ/Λ ~ 10^−5^. This means that the common approach of the LIL extension^32^, which consists in varying the incidence angle would require very precise angular adjustements of the order of Δ*θ* ~ 10^−4^ deg. Such small values are hard or even impossible to accurately realize by standard components utilized in LIL. An alternative to tiny angle variations is the use of small wedge angle prisms^29^, however this method significantly restricts the range of achievable periods and reduces the general fabrication flexibility.

To overcome the described difficulty we propose a solution based on the inhomogeneity of interference patterns produced by two point light sources, see Fig. 1. The inhomogeneity consists in a slow growth of the distance between the curved fringes (Fig. 1) with the change of the surface coordinate *x*, which can be used to replace a precision-demanding incidence angle adjustment Δ*θ* with a precision-tolerant sample shifting along the coordinate *x* between two consequent LIL exposures.

Denote the distance between point sources as *D*, and the distance from the source to the resist plane as *H*. The period of the quasi-periodic pattern from these sources shown in Fig. 1 in the central region near the symmetry plane *x* = 0 when $|x|\ll ({D}^{2}+4{H}^{2})/\sqrt{5{D}^{2}+3{H}^{2}}$ depends quadratically and slowly on *x* as follows (for details see Supplementary Materials):

3 $$\Lambda (x)\approx {\Lambda }_{0}+\frac{12\lambda {H}^{2}}{D{({D}^{2}+4{H}^{2})}^{3/2}}{x}^{2}$$

Here ${\Lambda }_{0}=\lambda \sqrt{{D}^{2}+4{H}^{2}}/2D$ is the desired grating period. Thus, a sample shift by Δ*x* between two identical exposures would result in a superposition of two periodic patterns with the ratio ΔΛ/Λ_0_ = *C*Δ*x*^2^, where *C* = 24*H*^2^/(*D*^2^ + 4*H*^2^)^2^ in the central region of the sample. Taking the values *D* = 69.3 cm, *H* = 32.3 cm and the wavelength *λ* = 442 nm of the He-Cd laser used in our experimental setup permits to attain Λ_0_ = 300 nm, and the substitution into the above formulae yields *C* ~ 3•10^−4^ cm^−2^. This small value of the constant *C* allows reaching extremely small ratios down to ΔΛ/Λ_0_ ~ 10^−9^ using standard manual linear translation stages with the smallest scale division of 20 *μ*m. The only modification required in the LIL setup is an addition of such translation stage to the sample support.

**Figure 2:**
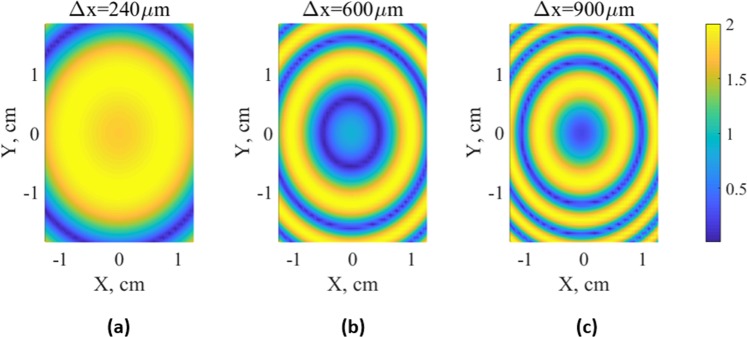
2D macroscopic envelope function (moiré pattern) of 1D variable-depth gratings obtained using the two-exposure process sketched in Fig. 1 for different sample shifts Δx = 240, 600 and 900 *μ*m. The nanoscopic grating period Λ = 300 nm at any point of the surface and the grating lines are oriented vertically. The dimensions of the X-Y simulation area correspond to the real sample size.

Superposition of interference patterns shown in Fig. 1 yields the desired beats. Figure 2 shows simulations of these beats, also called moiré patterns, for different sample shifts of 240 *μ*m, 600 *μ*m, and 900 *μ*m at realistic centimeter scale substrate dimensions. With the increase of the sample shift Δ*x* the ratio ΔΛ/Λ_0_ grows at any point of the surface, and the envelope function oscillates faster. The elliptical shape of the moiré pattern is due to the fact that the period Λ grows in any direction from the center as seen in Fig. 1, but at a different speed. The variations of Λ are enough to generate macroscopic moiré pattern, but at the same time they are negligibly small in comparison with the average grating period (at the corner of the sample the period changes by  ~ 0.1% or 0.3 nm only). To cover all possible grating depths from zero to the maximum value only one moiré ellipse is sufficient, so the sample shift Δ*x* = 240 *μ*m was chosen for the fabrication.

### Materials and fabrication

**Figure 3:**
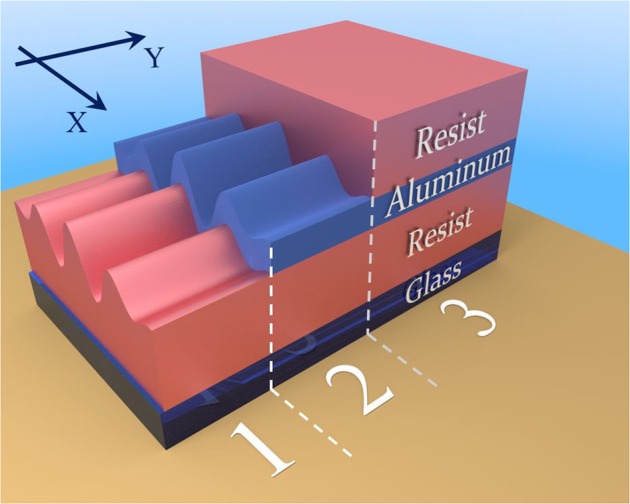
Schematic view of the three fabrication steps of a metallic variable-depth grating (1-resist deposition and lithography process; 2-grating metallization; 3-final resist deposition).

The sample designed for measurements in transmission is sketched in Fig. 3: it is the symmetric “insulator-metal-insulator” (IMI) structure on a glass support. The continuous metallic variable depth grating is sandwiched between two dielectric claddings. The geometry in Fig. 3 is divided by white dashed lines into three sections illustrating the main stages of sample fabrication.

In the first step, microscope glass slides BK7 3.7 × 2.5 cm × cm, used as a transparent substrate, were cleaned following a three-step wet bench procedure: 15 min in an acetone ultrasonic tank, 15 min in an ethanol ultrasonic tank and 10 min in a tank with pure deionized water. Clean glass substrates were then dried under a nitrogen stream. For the subsequent lithography, these substrates were spin-coated with the positive photoresist Shipley S1805 and soft-baked in an oven at 60 °C for 1 min to harden the resist, evaporate the solvent and improve the adhesion. The resist was diluted in ethyl lactate in order to control the layer thickness. A continuous He-Cd laser with the wavelength *λ* = 442 nm and effective power *P* = 500 *μ*W was utilized for the LIL. Photosensitive samples were pre-exposed homogeneously for 20 s at 250 *μ*W, allowing to use the resist in its linear regime. In the LIL process, two equal exposures for 15 s at 500 *μ*W each were conducted, with a sample shift of Δ*x* = 240 *μ*m between them. After a 4 s development and subsequent drying in a nitrogen stream, a dielectric variable depth grating of an average thickness of 250 nm is obtained, as sketched in Fig. 3, step 1.

**Figure 4:**
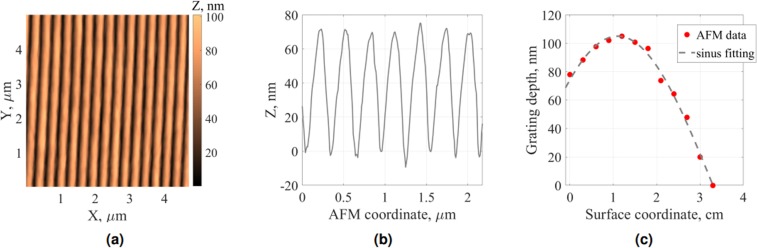
AFM data of the metallized grating (second fabrication step in Fig. 3) in the region of depths ≈7  nm: (**a**) top view and (**b**) cross-section perpendicular to grating grooves; (**c**) AFM-measured grating depth (red dots) as a function of surface coordinate and phenomenological sinus fitting of experimental data (gray dashed line).

In the second step the clean surface of the dielectric grating was metallized with aluminum (metallic thickness *h* = 17 nm) in a physical vapor deposition process (PVD) by sputtering of an aluminum target. After that AFM measurements were made along a central line of the moiré pattern along the *X* direction of the sample (see Fig. 2a). An example of an AFM-measured surface topology for a particular sample zone is presented in Fig. 4a for a top view and Fig. 4b for a cross-section. The experimentally measured grating depth as a function of the substrate coordinate on the mentioned line is shown in Fig. 4c. This dependence can be well fitted by a sinusoidal function, though the exact dependence is more complicated.

In the third and final fabrication step, the second resist layer of an average thickness of 690 nm was deposited on top of the metallic layer by spin coating, in order to obtain a symmetrical IMI structure.

## Resonant Transmission by Apodized Gratings

An advantage of the described variable-depth grating fabrication approach is that instead of synthesizing series of samples of different depth, all possible depths in a specified range are present at once. This allows to study pure depth-dependent effects on a single sample, since all other geometrical and structural parameters remain fixed.

To study depth dependent resonant effects in the transmission spectrum, the UV-Vis-NIR spectrophotometer Agilent Cary 5000 was used. Measurements in transmission offer advantages over the conventional reflection technique since the detected signal is less noisy than the reflected one and appears as a bright spot on the dark background. Moreover, for measurements under normal incidence the experimental setup is significantly simpler as all the elements are aligned with the light source^33^. The sample was fabricated in a region of the central moiré’s ellipse, and had an area of about 2.3 cm length, such that all grating depths from zero to the maximum value of approximately 105 nm were covered at least once. Due to the large longitudinal range of the height variation, an accurate probing of different heights was achievable even with unfocused beams, allowing the use of a 1 mm diameter beam for these measurements. The sample was fixed normally to the incident light and transmission spectra for both polarizations TE and TM were recorded along the line of AFM measurements for Fig. 4c. All the data was put together to visualize the resonant behavior in form of 2D depth/wavelength resolved maps.

**Figure 5:**
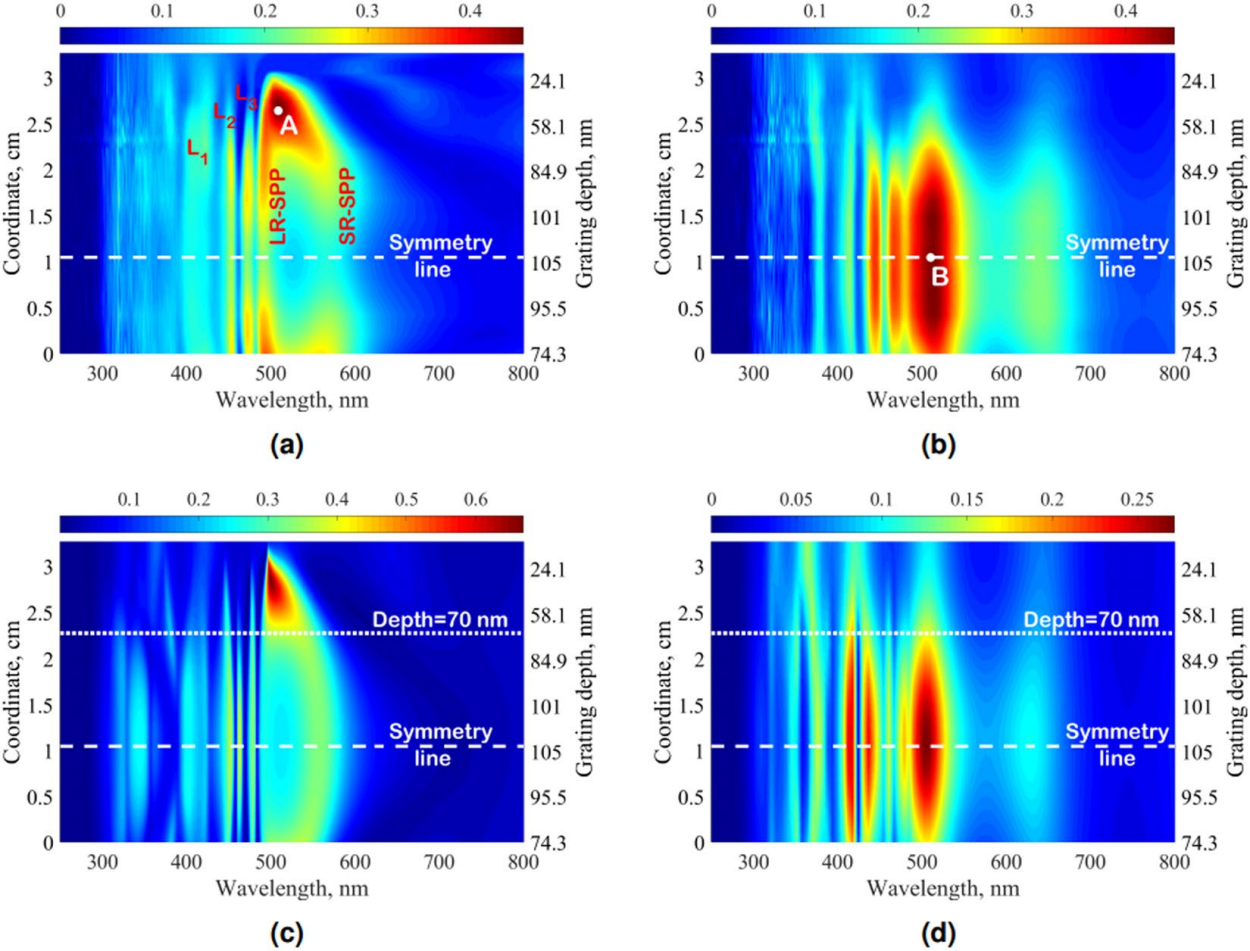
Normal transmission of the variable depth grating in VR for a depth range of 0–105 nm; (**a**) TM polarization, experimental data, (**b**) TE polarization, experimental data, (**c**) TM polarization, numerical simulation, (**d**) TE polarization, numerical simulation. The dotted horizontal lines in (**c**,**d**) indicate a grating depth of 70 nm, which was used for the angle-resolved transmission measurements in Fig. 8a,c.

The maps for the two polarizations in Fig. 5a,b demonstrate continuous variations of the visible range spectrum with the grating depth. Both figures have their symmetry line (marked by a dashed white line) at an *x* position of about 1.1 cm which is due to the symmetrical behavior of the grating depth envelope, see Fig. 4c. This symmetry underlines the fabrication quality, although for the study of the resonant transmission only one region with each depth is sufficient and will be considered in the following.

**Figure 6:**
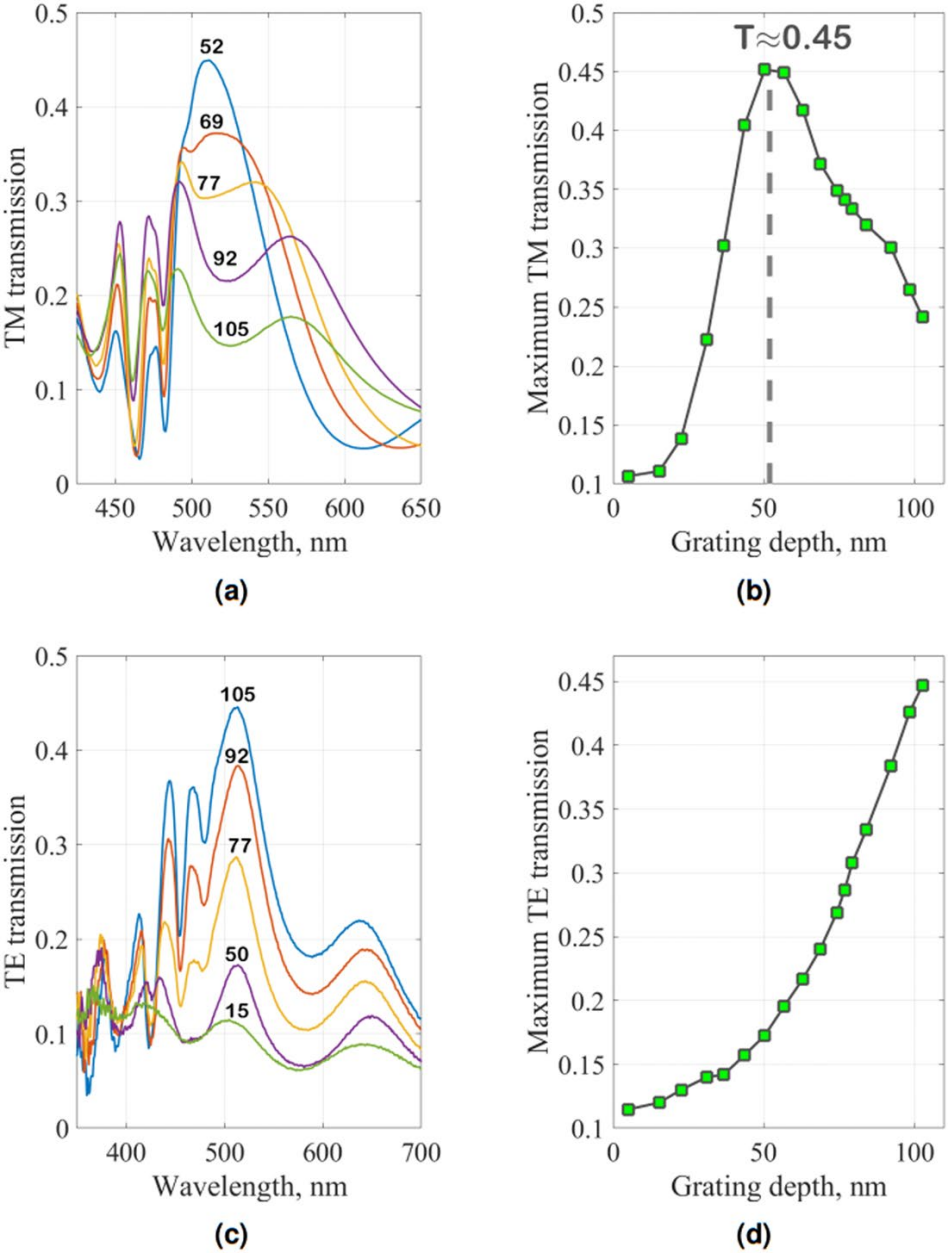
(**a**) Measured TM transmission spectra for grating depths of 52, 69, 77, 92, 105 nm; (**b**) Measured maximum plasmon-mediated transmission for all depths that are covered by the synthesized variable-depth grating, with the grey dashed vertical line indicating the optimal depth of 52 nm; (**c**) Measured TE transmission spectra for a set of depths: 15, 50, 77, 92, 105 nm; (**d**) Measured maximum TE transmission for all depths that are covered by synthesized variable-depth grating.

The most important feature in the TM polarization is the presence of plasmonic branches (denoted as LR-SPP and SR-SPP in Fig. 5a) corresponding to long- and short-range plasmonic modes respectively. The resonant transmission in this area appears due to the energy transfer from one metallic layer surface to the other by surface plasmon-polariton excitation. As is known^34^, the efficiency of this process depends strongly on the grating depth, which means that there should be an optimal depth with maximum plasmon-mediated resonant transmission. In our measurements this optimized grating depth exists (52 nm) and is marked by point *A* in Fig. 5a. The changes in transmission spectrum for different depths are demonstrated in Fig. 6a. Two plasmon resonance peaks are clearly visible for all depths, but the overall transmission efficiency changes dramatically. The measured maximum resonant transmission as a function of depth is plotted in Fig. 6b, demonstrating the experimentally optimized depth (vertical dashed line), with the maximum transmission of 45%.

Apart from the plasmonic effects, other spectral features can be investigated in the TM transmission plots. There are three vertical lines, denoted as *L*_1_ − *L*_3_ in Fig. 5a, which appear due to the waveguide mode excitation in the dielectric-metal-dielectric structure. Propagation constants of these modes are less sensitive to the grating depth in comparison to plasmonic modes, since their field distribution is localised primarily in the bulk of the dielectric resist layer, and are more affected by the thicknesses of the dielectric claddings. As all geometrical parameters of the fabricated sample are constant at any coordinate, except the depth, these modes manifest as the vertical bright transmission lines. The transmission efficiency of these modes reaches only half of the resonant plasmonic transmission (see Fig. 6a for grating depths of 69–77 nm, in the form of two minor peaks at *λ* ≈ 450 nm and *λ* ≈ 475 nm) that become less important when the depth approaches the optimal value of 52 nm.

**Figure 7:**
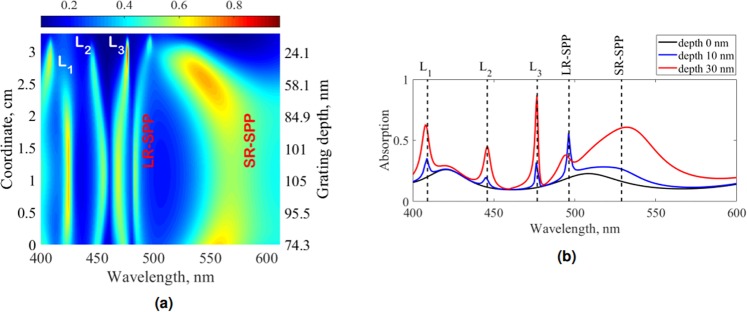
(**a**) Numerically calculated absorption map in TM polarization under normal incidence; (**b**) Numerically calculated absorption spectra for grating depths 0, 10 and 30 nm. Resonant wavelengths of plasmonic and waveguide modes denoted as vertical dashed lines are calculated in zero-depth limit for a planar lamellar structure.

The numerically calculated transmission (Fig. 5c), using the Generalized Source Method (GSM)^35^, reproduces all features of the experimental data including the positions of the vertical waveguide lines, a broad plasmon-mediated transmission area in the region of 500–600 nm wavelength, and the highest TM-transmission point A. Near this point the branches LR-SPP and SR-SPP are hardly distinguishable in both experimental and calculated data. Nevertheless, in a GSM-calculated absorption map presented in Fig. 7a these plasmonic lines are still clearly visible. The resonant wavelengths of plasmonic and waveguide modes (denoted in Fig. 7a as LR-SPP, SR-SPP and L_1_ − L_3_ respectively) can be predicted in the near-zero depth region (see Fig. 7b) by the 2 × 2 transfer matrix method^36,37^ for a planar lamellar waveguide with a thin metallic film inside the dielectric layer. Figure 7b shows that in the zero depth limit only broad Fabry-Pérot resonances are excited. However, with increasing grating depth the waveguide and plasmonic modes, being excited by the ±1 diffraction orders, appear.

**Figure 8:**
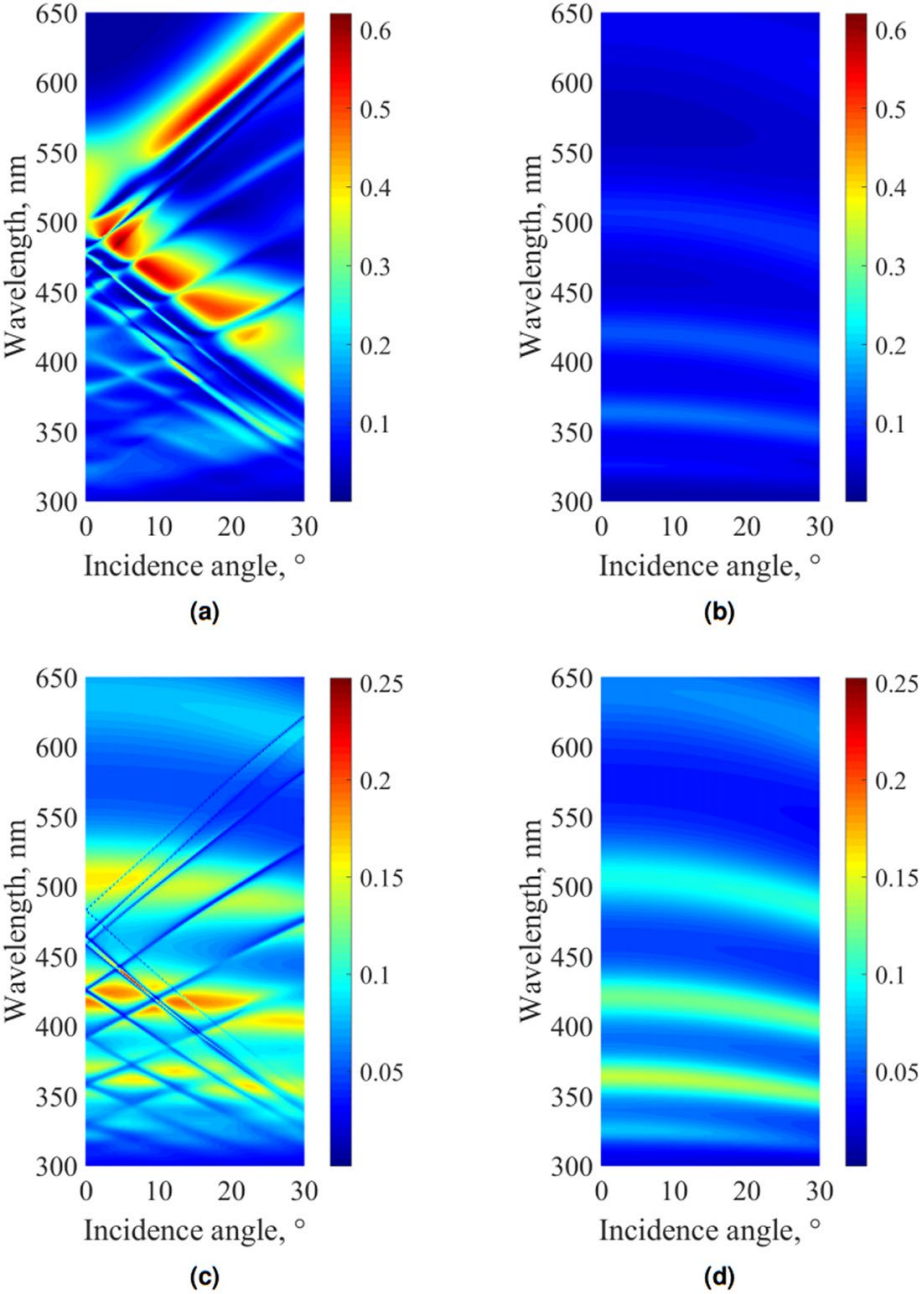
Numerically calculated angle-resolved TM transmission map in VR (**a**) for the grating depth of 70 nm (see horizontal dotted line in Fig. 5c) and (**b**) for planar geometry (grating depth equals zero); Numerically calculated angle-resolved TE transmission map in VR (**c**) for the grating depth of 70 nm (see horizontal dotted line in Fig. 5d) and (**d**) for planar geometry (grating depth equals zero).

Figure 8a represents an angle-resolved transmission map for an intermediate grating depth of 70 nm (horizontal white dashed line in Fig. 5c). This figure indicates firstly the wavelength splitting of waveguide and plasmonic modes and confirms that the plasmon-mediated resonant transmission area can be broad (50 nm for a depth of 70 nm in the wavelength range of 500–550 nm at normal incidence). Secondly, it also demonstrates that waveguide modes in TM polarization appear as bright lines of enhanced transmission on a dark background (compare with analogous Fig. 8c for TE polarization). In this dark background, Fabry-Pérot resonances can also be seen (as in Fig. 8b for planar geometry), but they are much weaker than the plasmon-mediated transmission.

Considering now the measurement results for TE polarization (Fig. 5b), one can see that the transmission also tends to grow with an increasing grating depth, analogously to the waveguide mode resonances in the TM case. The maximal experimentally measured TE transmission is located at the symmetry horizontal line (point B of the symmetry line in Fig. 5b). However, the influence of waveguide modes on energy transfer is different. As it can clearly be seen from the angle-resolved transmission map in Fig. 8c, waveguide modes in TE polarization appear as dark lines on a bright background. Consequently, TE waveguide modes do not contribute to the enhanced transmission, and in the experimental and calculated maps in Fig. 5b,d they exist as dark vertical lines on the bright background, as opposed to the TM case.

Figure 8d shows a simulated TE transmission map of the planar dielectric-metal-dielectric structure. This figure clarifies that enhanced TE transmission observed experimentally appears due to the Fabry-Pérot resonances and does not depend on waveguide modes and the grating period. The grating depth, however, still has a large influence on the transmission level, as it changes the effective optical characteristics of the whole layer. In analogy with the TM case, Fig. 6c demonstrates the variation of the transmission spectrum with a depth change: a bright background dominated by Fabry-Pérot resonances grows with minor transmission notches at the wavelengths of the waveguide resonances. Figure 6d shows the measured maximum TE transmission as a function of depth, it reveals that the maximum TE Fabry-Pérot-mediated transmission can reach the same absolute value of about 45% for the depth of 105 nm as the optimized TM plasmon-mediated transmission for the depth of 52 nm.

One can notice that despite a good reproduction of the experimental TE and TM spectral features in the numerical simulations, there are certain discrepancies in the absolute values: the maximum measured TM transmission (Fig. 5a, point A) is almost equal to the maximum measured TE transmission (Fig. 5b, point B), while numerical simulations predict a transmission in TM twice as big as in TE (Fig. 5c,d). This difference between theory and experiment arises from imperfections of the PVD: during this process the metallization growth rate varies from one sample region to another because of their different distance to the target center. The estimated inaccuracy of the metal deposition thickness of ≈10% gives a noticeable variation of transmission as the average metal thickness of 17 nm is very small.

## Conclusion

To conclude, this paper presents a novel LIL approach for the fabrication of varying-depth gratings. It is based on the well-known effect of beats of two waves of very close frequencies, and allows flexible and easy tuning of the modulation in a wide range in the form from microscopic to macroscopic moiré patterns. The only modification of a standard LIL setup is the addition of a linear stage to the sample support.

By means of the proposed method we have fabricated an apodized symmetric dielectric-metal-dielectric structure with adiabatically varying depth designed for resonances in the visible wavelength range. We experimentally confirmed the existence of an optimized grating depth, which appeared to be 52 nm for plasmon-mediated resonant TM transmission in our particular geometry. Additional resonant waveguide-mediated transmission of lower intensity also exists and tends to increase the efficiency with growing grating depth. In contrast, waveguide modes in TE polarization diminish the transmission. We have shown that in TE polarization wide regions of Fabry-Pérot-enhanced transmission exist, which are crossed by waveguide mode excitation resonances at certain wavelengths. Furthermore, an increase of the grating depth improves the transmission due to a Fabry-Pérot resonance excitation. All experimental features are in a good correspondence with rigorous numerical simulations.

The studied device can be used as a key element of plasmonic-based sensors^33^ with a possibility to optimize the operating grating depth for a given cover permittivity and get the highest possible transmission signal. The fabricated metallized and non-metallized variable depth gratings can also be used in optical security systems as for example Diffractive Optically Variable Image Devices (DOVIDs), since they demonstrate prominent angular- and polarization-dependent chromatic effects difficult to reproduce but visible by the naked eye^24^. Furthermore, adiabatically varying depth gratings can be commonly used in optical research for “experimental optimization” of depth-dependent effects.

## Supplementary information

Supplementary Materials
