# Supplementary material for: Systematic study of resonant transmission effects in visible band using variable depth gratings
Source: Sci Rep. 2019 Oct 17;9:14890. doi: 10.1038/s41598-019-51414-3 (PMC6797731; doi:10.1038/s41598-019-51414-3)
Supplement: Supplementary file 1 — Supplementary Materials [file 41598_2019_51414_MOESM1_ESM.pdf]

Supplementary Materials for Systematic study of resonant transmission effects in visible band using variable depth gratings

Andrei A. Ushkov[1,*], Alexey A. Shcherbakov[2], Isabelle Verrier[1], Thomas Kampfe[1], Yves Jourlin[1]

[1]Univ Lyon, UJM-Saint-Etienne, CNRS,
Institut d'Optique Graduate School, Laboratoire
Hubert Curien UMR 5516, F-42023 Saint-Etienne, France
[2]ITMO University, Saint-Petersburg, 190000, Russia
[*]andrei.ushkov@univ-st-etienne.fr

In this Supplementary Material we consider the interference by two point sources (see Fig. 1 of the Article). The difference in optical path between left and right beams in any point x on the resist surface is:

$$f(x) \equiv \Delta l(x) = \sqrt{(D/2+x)^2 + H^2} - \sqrt{(D/2-x)^2 + H^2} \tag{S1}$$

In the interval (x; x+dx) the phase shift between beams is:

$$\Delta\varphi = 2\pi/\lambda\,(f(x+dx) - f(x)) \tag{S2}$$

On the other hand, the same phase shift can be described in terms of the grating period Λ_1:

$$\Delta\varphi = 2\pi \cdot dx/\Lambda_1 \tag{S3}$$

Thus, the equations (S2-S3) give for Λ_1:

$$(f(x+dx) - f(x))/\lambda = dx/\Lambda_1 \Leftrightarrow \Lambda_1(x) = \lambda/f'(x) \tag{S4}$$

After some manipulations with (S1) and (S4) we get the Taylor decomposition of Λ_1:

$$\begin{aligned}\Lambda_1(x) = \frac{\lambda}{2D}\sqrt{D^2+4H^2} + 12\frac{\lambda}{D}\frac{H^2}{(D^2+4H^2)^{3/2}}x^2 \\ +16\frac{\lambda}{D}\frac{(5D^2+3H^2)}{(D^2+4H^2)^{7/2}}H^2x^4 + O(x^6)\end{aligned} \tag{S5}$$

So, for small enough x the period is a quadratic function of the coordinate. In this interval of x the second term should be much bigger than the third term on the right side of (S5):

$$12\frac{\lambda}{D}\frac{H^2}{\left(D^2+4H^2\right)^{3/2}}x^2 \gg 16\frac{\lambda}{D}\frac{\left(5D^2+3H^2\right)}{\left(D^2+4H^2\right)^{7/2}}H^2x^4$$
$$\Leftrightarrow |x| \ll \frac{D^2+4H^2}{\sqrt{5D^2+3H^2}} \sim 50\text{ cm}$$

Taylor decomposition (S5) allows also calculating the relative increment of Λ_1 with the coordinate x:

$$\frac{\Delta\Lambda\left(\Delta x\right)}{\Lambda_1\left(0\right)} = \Delta x^2\left[12\frac{\lambda}{D}\frac{H^2}{\left(D^2+4H^2\right)^{3/2}}x^2\right] / \left[\frac{\lambda}{2D}\sqrt{D^2+4H^2}\right] = C\Delta x^2$$

where

$$C = \frac{24H^2}{\left(D^2+4H^2\right)^2} \approx 3\cdot 10^{-4}\text{cm}^{-2}$$
